# Influenza Infection Rates, Measurement Errors and the Interpretation of Paired Serology

**DOI:** 10.1371/journal.ppat.1003061

**Published:** 2012-12-13

**Authors:** Simon Cauchemez, Peter Horby, Annette Fox, Le Quynh Mai, Le Thi Thanh, Pham Quang Thai, Le Nguyen Minh Hoa, Nguyen Tran Hien, Neil M. Ferguson

**Affiliations:** 1 MRC Centre for Outbreak Analysis and Modelling, Department of Infectious Disease Epidemiology, Imperial College London, London, United Kingdom; 2 Oxford University Clinical Research Unit - Wellcome Trust Major Overseas Programme, Hanoi, Vietnam; 3 National Institute of Hygiene and Epidemiology, Hanoi, Vietnam; Erasmus Medical Center, Netherlands

## Abstract

Serological studies are the gold standard method to estimate influenza infection attack rates (ARs) in human populations. In a common protocol, blood samples are collected before and after the epidemic in a cohort of individuals; and a rise in haemagglutination-inhibition (HI) antibody titers during the epidemic is considered as a marker of infection. Because of inherent measurement errors, a 2-fold rise is usually considered as insufficient evidence for infection and seroconversion is therefore typically defined as a 4-fold rise or more. Here, we revisit this widely accepted 70-year old criterion. We develop a Markov chain Monte Carlo data augmentation model to quantify measurement errors and reconstruct the distribution of latent *true* serological status in a Vietnamese 3-year serological cohort, in which replicate measurements were available. We estimate that the 1-sided probability of a 2-fold error is 9.3% (95% Credible Interval, CI: 3.3%, 17.6%) when antibody titer is below 10 but is 20.2% (95% CI: 15.9%, 24.0%) otherwise. After correction for measurement errors, we find that the proportion of individuals with 2-fold rises in antibody titers was too large to be explained by measurement errors alone. Estimates of ARs vary greatly depending on whether those individuals are included in the definition of the infected population. A simulation study shows that our method is unbiased. The 4-fold rise case definition is relevant when aiming at a specific diagnostic for individual cases, but the justification is less obvious when the objective is to estimate ARs. In particular, it may lead to large underestimates of ARs. Determining which biological phenomenon contributes most to 2-fold rises in antibody titers is essential to assess bias with the traditional case definition and offer improved estimates of influenza ARs.

## Author Summary

Each year, seasonal influenza is responsible for about three to five million severe illnesses and about 250,000 to 500,000 deaths worldwide. In order to assess the burden of disease and guide control policies, it is important to quantify the proportion of people infected by an influenza virus each year. Since infection usually leaves a “signature” in the blood of infected individuals (namely a rise in antibodies), a standard protocol consists in collecting blood samples in a cohort of subjects and determining the proportion of those who experienced such rise. However, because of inherent measurement errors, only large rises are accounted for in the standard 4-fold rise case definition. Here, we revisit this 70 year old and widely accepted and applied criterion. We present innovative statistical techniques to better capture the impact of measurement errors and improve our interpretation of the data. Our analysis suggests that the number of people infected by an influenza virus each year might be substantially larger than previously thought, with important implications for our understanding of the transmission and evolution of influenza – and the nature of infection.

## Introduction

Each year, seasonal influenza is responsible for about three to five millions severe illnesses and about 250,000 to 500,000 deaths worldwide [1]. These epidemics can generate important economic losses due to high levels of worker absenteeism as well as a saturation of emergency services at the peak of the epidemic [1]. In addition, avian or swine influenza viruses occasionally adapt to humans and generate influenza pandemics like in 1918, 1957, 1968 and 2009, sometimes with catastrophic consequences like in 1918, when 20 to 50 million people died worldwide.

Appropriate assessment of the epidemiological characteristics of the influenza virus is important to guide control policies. In particular, this requires being able to track the number of influenza cases with severe clinical outcomes (*i.e.* the tip of the severity pyramid) as well as the total number of people infected by an influenza virus (*i.e.* the base of the severity pyramid). For example, the case fatality ratio (proportion of influenza cases who die) is a key measure of severity that informs decision making during influenza pandemics, and takes the number of influenza related death as numerator and the number of influenza cases as denominator. Estimates of infection attack rates are also essential for characterizing the spread of the virus in human populations in order to predict epidemic trajectory, the potential impact of control measures such as social distancing measures, and the likelihood and magnitude of subsequent epidemics arising from continued circulation of the same virus [2], [3].

Although it is usually possible to estimate the number of severe influenza cases from sentinel surveillance (e.g. based on data collected at medical practices, clinics or hospitals), it is much harder to estimate the total number of people infected by an influenza virus. First, a substantial proportion of influenza infections are asymptomatic [4], [5]. Second, among those with symptoms, only a proportion seek healthcare; and this proportion may vary from season to season or even during the course of an epidemic. Last, Influenza-Like-Illness (ILI) symptoms are not specific to influenza. So, a substantial proportion of patients consulting for ILI may not have been infected by an influenza virus.

Serological studies have become the gold standard approach for estimating influenza infection attack rates due to the difficulty of estimating infection rates by other means. Although cross-sectional serological surveys can provide valuable and timely information, paired blood samples collected before and after an epidemic in a cohort of individuals is the optimal approach for precisely assessing infection rates. The haemagglutination-inhibition (HI) assay remains the most commonly used approach for detecting serological evidence of recent influenza infection [6]–[12]. The assay detects the presence of antibodies that prevent the haemagglutinin protein of the influenza virus from agglutinating red blood cells [13], [14]. For each serum sample, antibody titers are expressed as the reciprocal of the highest serum dilution that can still prevent a fixed concentration of virus from agglutinating red blood cells. A rise in antibody titers between the first and second blood is taken as a marker of infection. However, because the procedure is susceptible to measurement errors, a 2 fold rise (that is a 1-dilution increase) is usually considered as insufficient evidence for infection. Seroconversion is therefore typically defined as a 4-fold rise (*i.e.* a 2-dilutions increase) or more in antibody titers. This ad-hoc rule became established when these methods were first developed and is now widely adopted [15], [16]. In the meantime, however, statistical methods for addressing measurement errors have made substantial progress. In particular, there is now an extensive body of literature on methods to ensure that the presence of measurement errors does not bias estimates of key parameters of interest. Given these developments, it is timely to revisit the way serological data are interpreted.

Central to the traditional approach to analyzing serological data is the belief that data about 2-fold rises provide no information since such increases can be caused by frequent measurement errors. This concern about measurement errors is certainly relevant when trying to make specific diagnoses for individual cases. For example, one may be averse to the risk of false positives; but less so to the risk of false negatives. However, estimating infection attack rates at the population level is a very different aim from setting up a specific diagnostic tool, and may benefit from a different use of the data.

First, it is important to note that estimating infection attack rates is not just a matter of specificity (*i.e.* ensuring that subjects satisfying the diagnostic definition of infection were indeed infected by an influenza virus) but also a matter of sensitivity (*i.e.* ensuring that all subjects infected are diagnosed as such). An approach that favours specificity over sensitivity may lead to underestimating infection attack rates.

A second important observation is that, even in a context of frequent 2-fold errors, data about 2-fold rises may still be informative. Consider for example a situation where all individuals exhibit a 2-fold rise during the season: such a pattern cannot be explained by measurement error alone since measurement errors are made *both* at baseline and post-epidemic and should be about equally distributed provided the sample size is sufficiently large.

Here, we explore how modern statistics for the analysis of data with measurement errors can change and improve our interpretation of serology. We present a new method to quantify errors in the measurement of antibody titers and to estimate the true distribution of paired serological measurements corrected for measurement errors. The methodology is applied to data collected in a cohort study conducted in Vietnam between 2007 and 2009.

## Results

### Measurement errors

**Figure 1:**
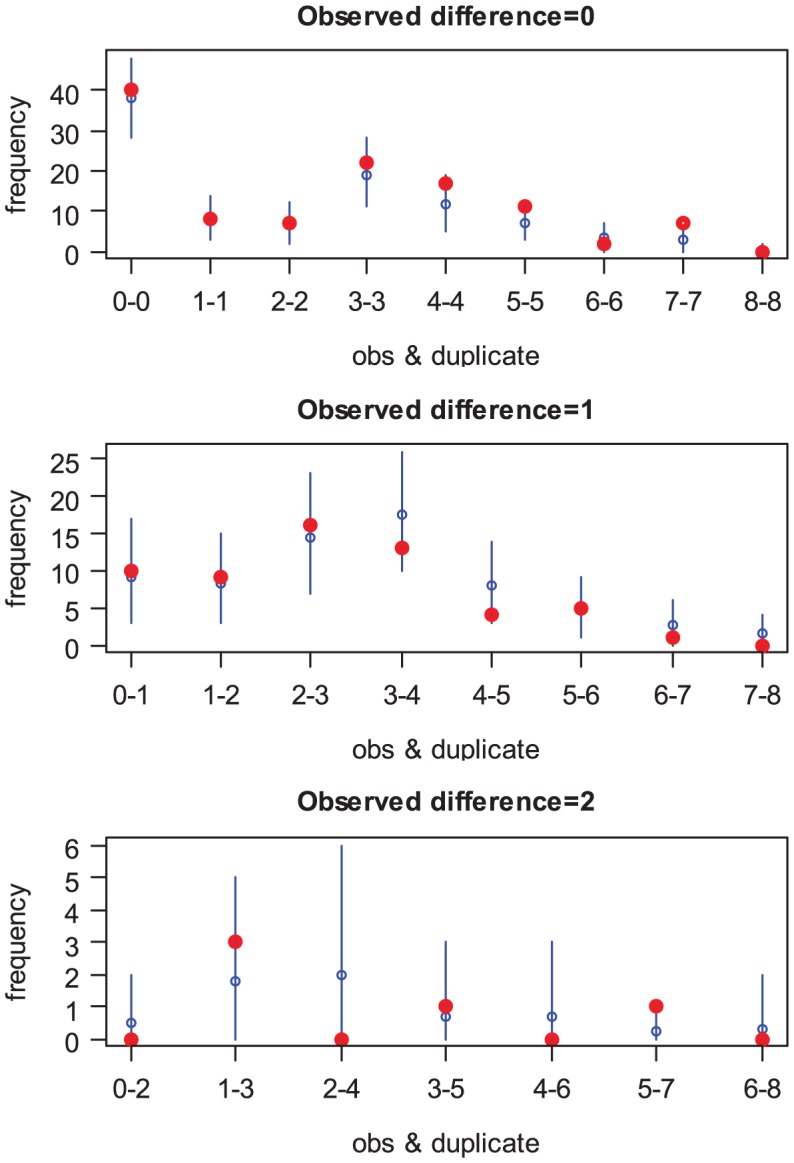
Fit of the model to data on replicate measurements. Observed (red point) and expected (mean: blue point/95% CI: blue bar) number of pairs (observed AT level, replicate AT level). Pairs are sorted by panel according to the number of dilution difference between the observed and the replicate measurement.

We estimate that the 1-sided probability of a 2-fold error was 9.3% (95% CI: 3.3%, 17.6%) when the true antibody titer was below detection levels, rising to 20.2% (95% CI: 15.9%, 24.0%) otherwise (posterior probability that latter larger than former: 98.7%). There was a satisfying fit of the model to replicate measurement data (Figure 1). The model where measurement errors were independent of true antibody titers failed to fit the data (Figure S2 and Supplementary Material).

### Distribution of true paired serology

**Figure 2:**
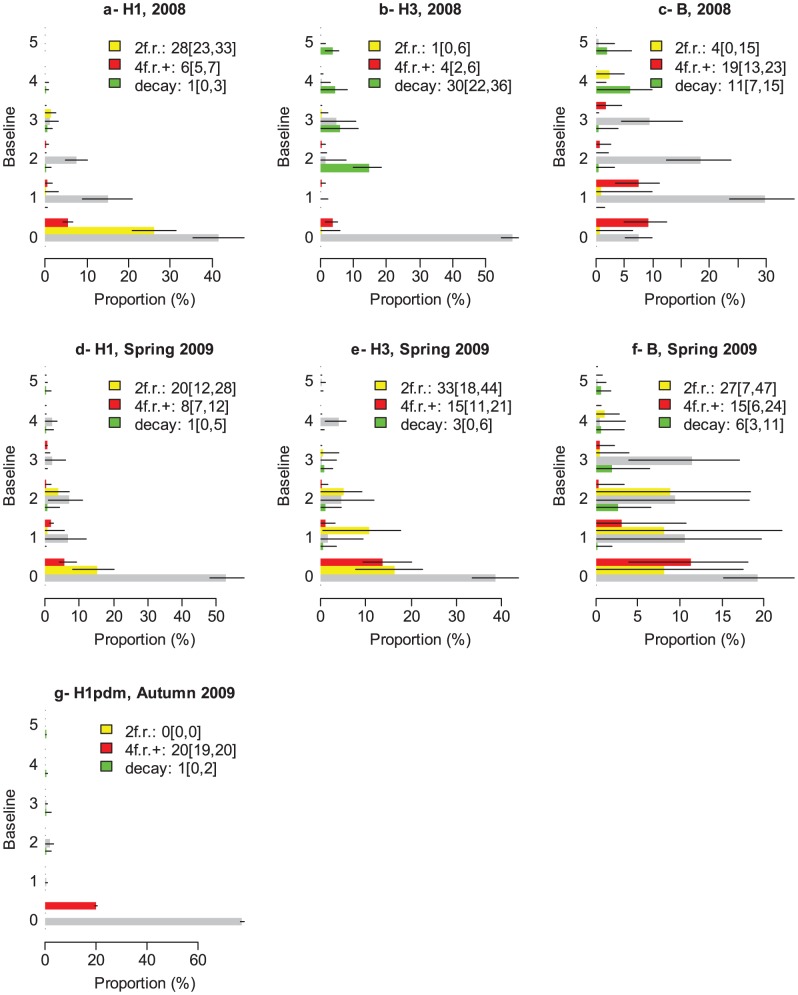
Distribution of paired serology, corrected for measurement errors as a function of season (2008, Spring 2009, Autumn 2009) and subtype (H1N1, H3N2 and B) (in Autumn 2009, subtyping was only conducted for H1N1pdm09). In each panel, individuals are sorted by baseline AT levels on the y-axis. For a given baseline, the grey bar indicates the expected proportion of individuals with post AT level equal to baseline AT level; the yellow bar indicates the proportion with a 2 fold rise (2f.r.); the red bar indicates the proportion with a 4 fold rise or more (4f.r.+); the green bar indicates the proportion with a decay. The black thin lines give the 95% CI. The legend gives the mean [95% CI]. **A**: H1N1, 2008. **B**: H3N2, 2008. **C**: B, 2008. **D**: H1N1, Spring 2009. **E**: H3N2, Spring 2009. **F**: B, Spring 2009. **G**: H1N1pdm09, Autumn 2009.

Figure 2 summarizes the distribution of paired serology, corrected for measurement errors for the different seasons (2008, Spring 2009, Autumn 2009) and subtypes (H1N1, H3N2 and B). A range of observations can be made.

**Figure 3:**
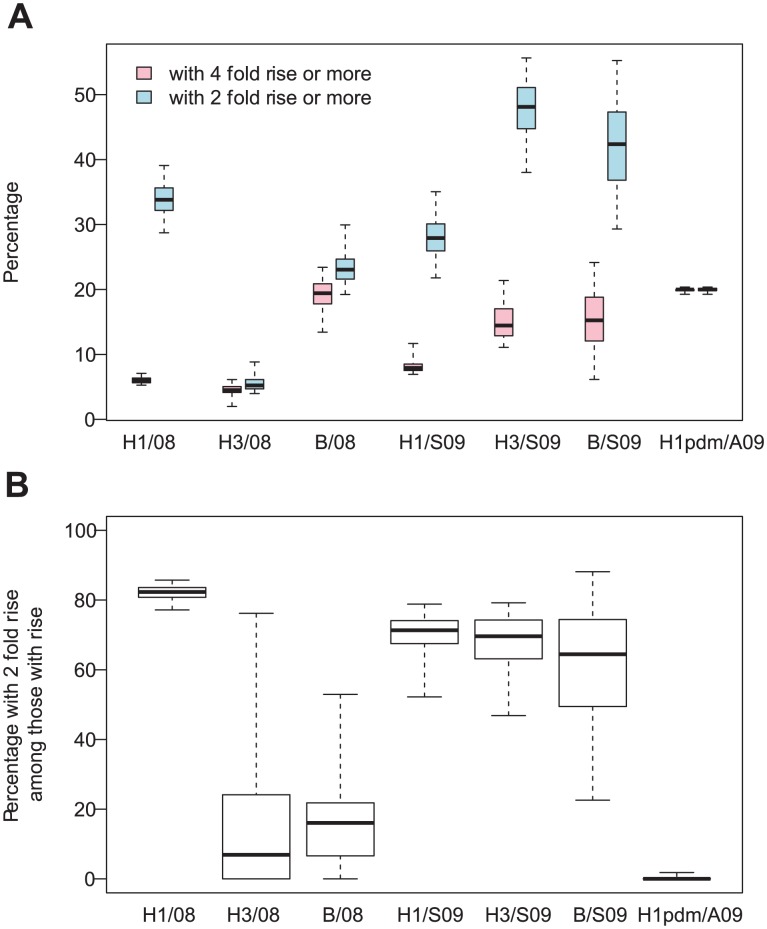
Increases in antibody titers. **A**: Posterior distribution of the percentage of subjects with a 4 fold rise or more in AT (pink) and with a 2 fold rise or more in AT (blue) for the different subtypes and the different seasons (2008 (08), Spring 2009 (S09), Autumn 2009 (A09)). **B**: Posterior distribution of the percentage of subjects with a 2 fold rise in AT among those with a rise in AT. Boxplots give percentiles 2.5%, 25%, 50%, 75%, 97.5% of the distribution.

The first observation concerns 2-fold rises in antibody titers between baseline and post serology (yellow bars). Such increases are usually ignored in analyses because 2-fold errors are common. In some instances, like for example subtypes H3N2 and B in 2008 and H1N1pdm09 in Autumn 2009, 2-fold rises appeared negligible and at levels that could be generated by measurement errors alone, since 0 was within the 95% CI of the estimated proportion of subjects having a 2-fold rise (Figures 2B, 2C, 2G). In other instances, however, the proportion of individuals experiencing a 2-fold rise ranged from 20% to 33% with lower bounds of the 95% CIs above 0 (range: 7%–23%), indicating that these rises cannot be solely explained by measurement errors. Assuming that most of these 2-fold rises were due to infection, our estimate of infection attack rates 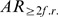 for H1N1 in 2008 and H1N1, H3N2 and B in Spring 2009 would be dramatically higher than traditional estimate 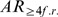 based on 4-fold rises or more (Figure 3A). So, even if only a proportion of the 2-fold rises were due to influenza infections, the traditional estimate 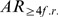 might still represent a substantial underestimate of the true infection attack rates

The fact that 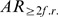 and 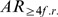 were very similar for H3N2 and B in 2008 and virtually identical for H1N1pdm09 in Autumn 2009 (Figure 3A) highlights important heterogeneities in the way antibody titers increase by season/subtype (Figure 3B). For example, for H1N1pdm09 in Autumn 2009, almost all those experiencing a rise in antibody titers exhibited a 4-fold rise or more; but for H1N1 in 2008, most of those experiencing a rise only had a 2-fold increase. The absence of a simple linear relationship between 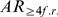 and the proportion of 2-fold rises suggests that the standard approach of inflating 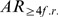 by a fixed proportion (generally equal to the proportion of PCR positive cases who do not seroconvert; around 10–20%) to get corrected estimates of infection attack rates may be inappropriate. Rather, corrections might have to be applied on a season-to-season and subtype-to-subtype basis.

The last notable observation is that decay in antibody titers is observed. For example, 30% (95% CI: 22, 36) of individuals exhibited a decay for subtype H3N2 in 2008.

### PCR positive cases

**Figure 4:**
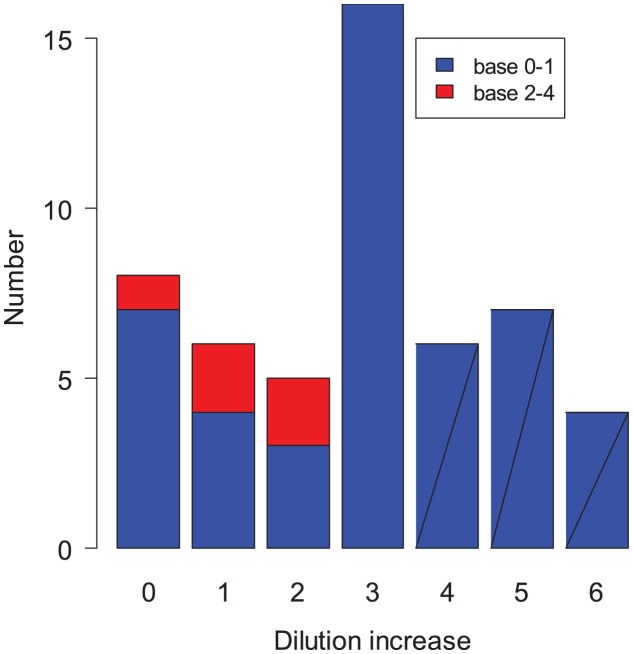
Distribution of observed increase in PCR positive cases as a function of baseline. Individuals with a low antibody titer baseline (0–1) are in blue; those with a higher baseline (2–4) are in red.

Figure 4 shows the observed rise in antibody titers for PCR positive cases. Twenty seven percent of these cases experienced no rise or only a 2-fold rise in titer during the season. This again suggests that the case definition of a 4-fold rise or more may underestimate attack rates by at least 27%. PCR positive cases with low baseline titers experienced an average increase significantly larger than those with higher baseline titers (p = 0.026) (Figure 4) [17], [18].

### Cross-reactivity between subtypes

Simulations were run to test the hypothesis of an absence of cross-reactivity between subtypes H1N1, H3N2 and B in 2008 and Spring 2009 (see Supplementary Material). We found that there was good adequacy between the data and patterns that would be obtained in the absence of cross-reactivity. The hypothesis of an absence of cross-reactivity could therefore not be rejected (Figure S3).

### Model fitting

**Figure 5:**
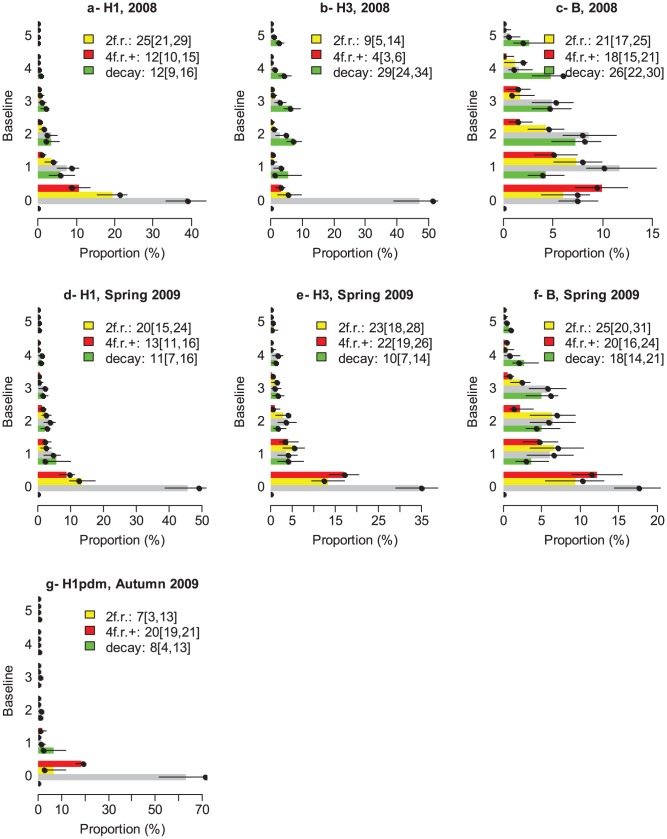
Model adequacy to the data. Distribution of “observed” paired serology as predicted by the model (color bars) and as observed in the data (black point) as a function of season (2008, Spring 2009, Autumn 2009) and subtype (H1N1, H3N2 and B). In each panel, individuals are sorted by baseline AT levels on the y-axis. For a given baseline, the grey bar indicates the expected proportion of individuals with post AT level equal to baseline AT level; the yellow bar indicates the proportion with a 2 fold rise (2f.r.); the red bar indicates the proportion with a 4 fold rise or more (4f.r.+); the green bar indicates the proportion with a decay. The black thin lines give the 95% CI. The legend gives the mean [95% CI]. **A**: H1N1, 2008. **B**: H3N2, 2008. **C**: B, 2008. **D**: H1N1, Spring 2009. **E**: H3N2, Spring 2009. **F**: B, Spring 2009. **G**: H1N1pdm09, Autumn 2009.

Figure 5 compares the distribution of observed paired serology as observed in the data (black point) and as predicted by the model. Model fit was satisfactory.

### Simulation study

**Figure 6:**
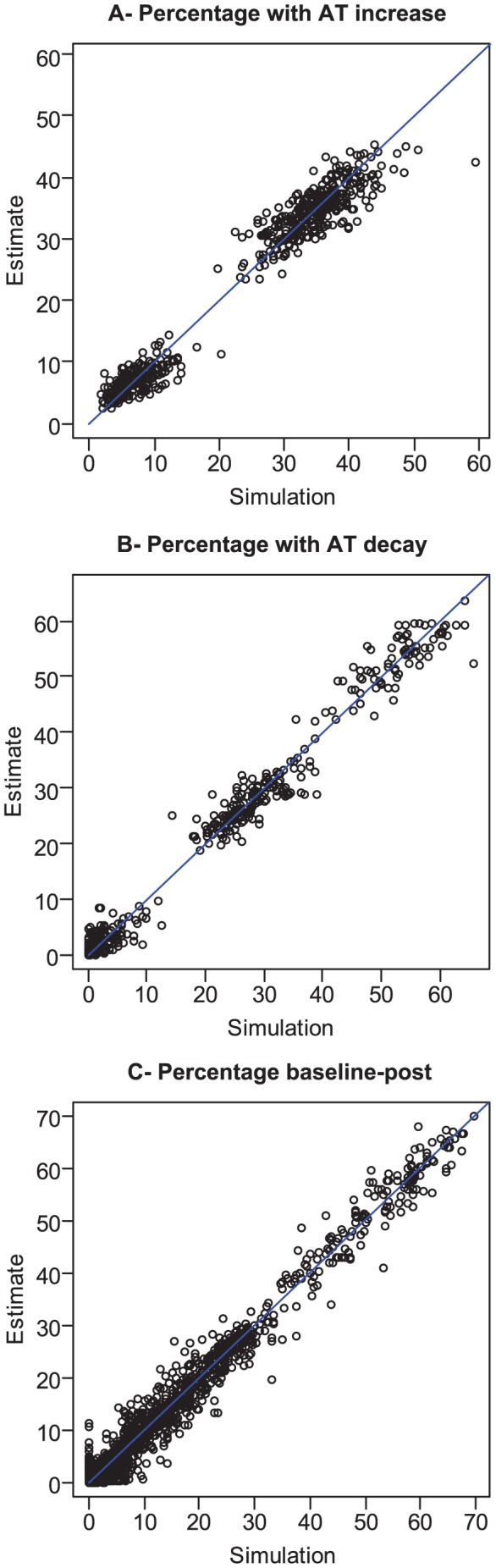
Performance of the method to reconstruct the true distribution of paired serology. Eighty datasets are simulated with known parameters (see Methods). **A**: Estimated percentage of subjects with an increase in antibody titers as a function of the true percentage in the simulated dataset. **B**: Estimated percentage of subjects with a decay in antibody titers as a function of the true percentage in the simulated dataset. **C**: Estimated probabilities characterizing jointly baseline AT level and the change in AT level during the epidemic _ similar to those presented in Figure 1 _ as a function of the true probability in the simulated dataset.

In a simulation study, we found that estimates of parameters characterizing measurement errors were unbiased (Table 1), as well as those characterizing the selection process (Table S2). We also found that estimates of the proportion of subjects with an antibody titer increase (empirical absolute bias: 0.1%), of the proportion of subjects with an antibody titer decay (empirical absolute bias: 0.0%) and of the probabilities characterizing jointly baseline antibody titers and the change in antibody titers during a season (empirical absolute bias: 0.0%) were unbiased (Figure 6).

**Table 1:** Performance of the method to estimate parameters characterizing measurement errors.

	*p_0_*	*p_1_*	*ε*
**Simulation value**	9.0%	20.0%	0.50%
**Mean estimate (SD)**	9.5% (4.1%)	19.8% (2.3%)	0.065% (0.21%)

*p0*: probability of a 1-sided 1-dilution error if true AT level is  = 0.

*p1*: probability of a 1-sided 1-dilution error if true AT level is >0.

*ε*: probability that measurement goes wrong and that observed AT level is Uniformly drawn in (0,…,K).

Eighty datasets are simulated with known parameters (see Methods). The table gives the simulation value of parameters and the mean (standard deviation) of estimates.

### Age-specific patterns

Our statistical model describes the distribution of paired serology across all subjects. However, since we infer true paired serology for each individual, it is possible to reconstruct a posteriori the distribution of true paired serology for the different age groups. The age-specific distributions for true paired serology are presented in Figure S4. Interesting differences can be noticed between age groups. For example and consistent with the literature, for H1N1pdm09 in Autumn 2009, the proportion of 4-fold rises falls from 39% (95% CI: 37%, 39%) in <18 y.o. to 15% (95% CI: 15%,16%) in 18–48 y.o. and 8% (95% CI: 7,9) in >48 y.o. For H3N2 in 2009, the decay in antibody titers was more important among <18 y.o. (53%; 95% CI: 38%, 65%) than among older age groups (25%, 95% CI 19%, 30% for 18–48 y.o. and 18%, 95% CI 12, 22 for >48 y.o.). For H3N2 in Spring 2009, although the proportions of 4-fold rises were similar across age groups, our analysis suggests that the proportion of 2-fold rises may have been higher among <18 y.o (43%, 95% CI: 23, 58) than in other age groups (30%, 95% CI 17%, 41% for 18–48 y.o. and 27%, 95% CI 13, 38 for >48 y.o.). We find that, for each age group, there is a satisfying adequacy between the observed distribution of paired serology and that predicted by the model (Figure S5).

## Discussion

In this paper, we have revisited the traditional interpretation of paired serological measurements of influenza antibody titers. Until now, data on 2-fold rises have been largely ignored because of the belief that measurement errors made them unreliable. Although this may be a valid concern if the aim is to get a specific diagnosis for individual cases, we argue that this is less so when the objective is to interpret antibody titer variations at the population level. We have shown that it is possible to quantify measurement errors, and to reconstruct the distribution of paired serology corrected for measurement errors. Our method gave unbiased estimates in a simulation study.

After correction for measurement errors for the Vietnamese data examined here, we found that for some seasons and subtypes the proportions of individuals with 2-fold rises in antibody titers was too large to be explained by measurement errors alone. Estimates of infection attack rates varied greatly depending on whether or not 2-fold rises were included. It is therefore important to determine the biological phenomenon that could cause such increases, in particular whether they are caused by exposure to influenza viruses.

A first hypothesis is that 2-fold titer increases are caused by infection by an influenza virus. In support of this hypothesis, it is clear that a proportion of virologically- or RT-PCR- confirmed influenza cases do not achieve a 4-fold rise in HI titer. This proportion was 27% in our dataset, similar to a large cohort of confirmed pandemic cases in the US [19]. However, past work has shown this proportion to be as high as 77% in people who have high pre-existing antibody titers [17], or as low as 10% in patients seeking medical care for pandemic H1N1 infection in 2009 [20]. It is clear that antibody titer changes following infection vary between individuals and are affected by factors including pre-existing titer and timing of serum collection. In particular, since there is an upper limit to antibody concentrations, individuals with high pre-existing titers are limited in their ability to generate 4-fold rises and may produce only a 2-fold titer increase in response to infection [15]. However, the analysis performed here shows that 2 fold titer changes are common even among individuals with low pre-existing titers. Antibody concentrations reach a peak 4–7 weeks after infection and then decay over a period of around six months to a plateau that is maintained for several years [21]. Although the profile of HA antibody decay is not well characterised, the probability of detecting 2- or 4- fold rises will vary with the interval following infection. However, in our data the longest interval between the peak transmission period and blood sampling was in season 3, when the proportion of 2-fold titer rises was lowest.

A second hypothesis is that 2-fold rises correspond to infection which is attenuated by mucosal or serological antibodies to homologous or heterologous strains, or by innate or cell mediated immunity. Antibody responses to inactivated influenza vaccines clearly demonstrate the potential for antigenic stimulation without active infection and the phenomenon of boosting of immunity in exposed yet uninfected individuals is well documented for other viruses (*e.g.* varicella zoster [22]).

A third hypothesis is that 2-fold rises are an artefact unrelated to influenza infection or exposure. Seasonal variation in titres independent of infection might result from the presence of non-specific inhibitors of agglutinination. For example, this could happen if the circulation of other viruses boosted the immune system, leading to small increases in all antibody titers. In such a scenario, one might expect the effect to be similar on the different subtypes. However, in 2007, a large proportion of individuals exhibited 2-fold increases for H1N1 but not for H3N2 or B, suggesting that this hypothesis is not strongly supported by the data.

It is also important to understand why 2-fold titers changes were prominent during some seasonal influenza epidemics but not during the pandemic. One possibility may be that there was greater antigenic mismatch for some seasonal strains because of unrecognised co-circulation of different influenza strains from those used as antigens in the HI assay. In this situation, anti-HA antibodies generated by infection have lower avidity for the HA of the assay virus. Conversely, original antigenic sin, where an infection results in an anamnestic response and the generation of antibodies directed towards an earlier infecting strain, might also explain 2-fold titer rises in response to infection [17]. In all these scenarios however, 2-fold increases would still represent infection by an influenza virus.

It is unlikely that 2-fold increases represent cross-reactivity of HI antibodies to strains of one subtype with strains of other subtypes. This is confirmed by our analysis that did not reject the hypothesis of an absence of cross-reactivity between subtypes.

It is therefore important for future work to determine if 2-fold titer increases represent infection, antigenic stimulation (attenuated infection), or artefact. If influenza infection rates are higher than currently recognised this might change our understanding of influenza transmission and of intra-host and inter-host immune mediated evolutionary pressures, and may have implications for the feasibility of control measures.

In the dataset examined here, 2-fold increases exceeded 4-fold increases for H1N1 in 2008 and H1N1, H3N2 and B in Spring 2009. There was no clear pattern with respect to subtype or strain. The seasonal H1N1 strain circulating in 2008 (A/Brisbane/59/2007) was antigenically distinct from those circulating previously (A/Solomon Islands/03/2006 and A/New Caledonia/20/1999-like), but this strain continued to circulate in Spring 2009. The seasonal H3N2 strain circulating in Spring 2009 (A/Perth/16/2009) was antigenically distinct from the 2007/8 strain (A/Brisbane/10/2007). H3N2 A/Perth/16/2009-like viruses have been difficult to propagate and we had difficulty propagating sufficient virus for the HI assays using A/Perth/16/2009-like viruses isolated from the cohort during the Spring 2009 season. We therefore used a virus isolated from a patient in Hanoi by the National Influenza Center, and propagated in eggs followed by MDCK cells (TX265M2E1) for undertaking HI testing of sera collected in Spring 2009. It is possible that the propagation in eggs this virus underwent might have resulted in some antigenic change, resulting in lower titers in the HI assay. National influenza surveillance data indicates that both influenza B lineages - Yamagata and Victoria- co-circulated during the study period, with the Yamagata lineage dominating in 2007 and 2008 and the Victoria lineage in 2009. For all HI assays, we used the same influenza B virus, which was isolated in 2008 and was characterized antigenically as Yamagata lineage-like, as with all influenza B viruses isolated from the cohort in 2008. While Yamagata viruses dominated the influenza B samples we collected in 2007 and 2008, the Victoria lineage was predominant in 2009. This may be a factor explaining the lower influenza B titer increases seen in that year. If heterogeneities in the proportion of 2-fold titer rises are largely attributable to a poor match between assay antigen and infecting virus, future seroprevalence and seroincidence surveys will need to use a greater diversity of antigens than typically used currently.

There are often strong age-related patterns in influenza serology. Ideally, we would therefore like to fit our statistical model independently for each age group. However, simulation studies indicate that the relatively small number of observations per age group would lead to relatively inaccurate estimates. We have therefore opted for an intermediate estimation strategy. Our statistical model fits a single distribution of true paired serology to all subjects; but since we infer true paired serology for each individual, we can reconstruct a posteriori the distribution of true paired serology for the different age groups. Even with such a conservative approach (*i.e.* it favours scenarios where the different age groups exhibit similar distributions), we were able to detect clear age-related patterns. In particular, it indicated that age may be another factor that influences the occurrence of a 2-fold rise. Larger sample sizes will be needed to investigate this possibility further.

The presence of relatively large proportions of individuals experiencing a 2-fold increase in antibody titers is not a peculiarity of the Vietnamese data examined here. Similar shifts were observed on data gathered by Cowling et al, with micro-neutralization assays for 2009 H1N1pdm09 influenza and on HI assays for seasonal influenza [23] (Figure S6).

It is well known that there may be substantial within- and between- laboratory variability in HI assays as well as in other serological assays such as virus neutralisation (VN) [24]. The level of intra-laboratory variations may depend on both the laboratory and the type of assay used [24]. Here, we have introduced an approach that allows controlling for within-laboratory variations. The only additional data needed compared with standard serological surveys is that replicate measurements are performed for a subset of subjects. These replicate measurements allow within-laboratory quantification of variation in assay performance. With this information, it is then possible to reconstruct the distribution of paired serology that is corrected for the estimated level of within-laboratory variations. Although our approach gives a better control on within-laboratory variation, it does not address the problem of between-laboratory variation. The use of standards in bioassays is critical for minimising the impact of the latter problem [24].

To conclude, while a 4-fold titer increase may be a highly specific diagnostic of infection by an influenza virus for individual cases, this criterion is less justifiable when the objective is to estimate community ARs. Our work shows that requiring a 4-fold titer increase may lead to ARs being substantially underestimated. More research is needed to determine what proportion of 2-fold rises are causally linked to exposure to influenza, and what proportion may be caused by other mechanisms. It will be important to determine whether the high proportion of 2-fold titer increases seen in the settings of Vietnam and Hong Kong [23] are also observed in other (e.g. temperate climate) settings.

## Materials and Methods

### Data

Samples were collected from a household-based cohort of 940 participants in 270 households in a single community in semi-rural northern Vietnam as previously described [5]. None of the participants had ever received influenza immunisation. Participants were under weekly active surveillance by village health workers for influenza-like-illness (ILI) and in the event of an ILI were asked to provide a nose and throat swab for detection of influenza RNA by reverse-transcription polymerase chain reaction. Participants were also asked to provide serial blood samples at times when national influenza surveillance data indicated that influenza circulation was minimal. The samples described here were collected over a period of three consecutive influenza seasons, from December 2007 through April 2010. The bleeding times were 1st–7th December 2007 (bleed 1), 9th–15th December 2008 (bleed 2), 2nd–4th June 2009 (bleed 3), and on the 3rd April 2010 (bleed 4). This provided three sets of paired samples either side of an influenza transmission season: 548 paired samples for season 1 (2008), 501 paired samples for season 2 (Spring 2009), and 540 paired samples for season 3 (Autumn 2009). In season 1, the influenza A virus strains detected in the cohort through ILI surveillance were A/H1N1/Brisbane/59/2007-like and A/H3N2/Brisbane/10/2007-like; in season 2, they were A/H1N1/Brisbane/59/2007-like and A/H3N2/Perth/16/2009-like; and in season 3, it was A/H1N1/California/7/2009-like. There was co-circulation of influenza B Yamagata lineage and Victoria lineage in both season 1 and season 2, with a predominance of Yamagata lineage in season 1 and Victoria lineage in season 2.

### Laboratory methods

Nasal and oropharangeal swabs were assessed by real-time reverse-transcriptase polymerase chain reaction (RT-PCR), according to WHO/USCDC protocols [25]. Influenza hemagglutination inhibition (HI) assays were performed according to standard protocols [WHO 2011 manual]. The seasonal influenza A viruses used were isolated from participants' swabs or from swabs taken from patients presenting in Ha Noi in the same season and propagated in embryonated hen's eggs or in MDCK cells. A reference antigen supplied by WHO (A/H1N1/California/7/2009-like) was used to assess season 3/pandemic sera. A single influenza B virus isolated from a participant during 2008 was used to assess serum for both the first and second seasons. The virus had a titer of 320 with B/Wisconsin/1/2010 (Yamagata) reference antisera and of <10 with B/Brisbane/60/2008 (Victoria) antisera. Each virus was first assessed for haemagglutination of erythrocytes from chickens, guinea pigs and turkeys then titrated with optimal erythrocytes. Serum was treated with receptor destroying enzyme (Denka Seiken, Japan) then heat inactivated and adsorbed against packed erythrocytes. Eight 2-fold dilutions of serum were made starting from 1∶10 and incubated with 4 HA units/25 µl of virus. Appropriate erythrocytes were added and plates read when control cells had settled. Virus, serum and positive controls were included in each assay. Pre- and post-season sera were tested in pairs. Each serum was tested in a single dilution series. The HI titre was read as the reciprocal of the highest serum dilution causing complete inhibition of RBC agglutination, partial agglutination was not scored as inhibition of agglutination. If there was no inhibition of HI at the highest serum concentration (1∶10 dilution) the titer was designated as 5. Only one sample had a titer >1280 and this was not adjusted. Replicate HI assay measurements were performed on a subset of samples from patients that seroconverted (i.e. 4-fold rise in titer) as well as some others that had titers ≥20 in both pre and post-season sera.

### Statistical analysis

**Figure 7:**
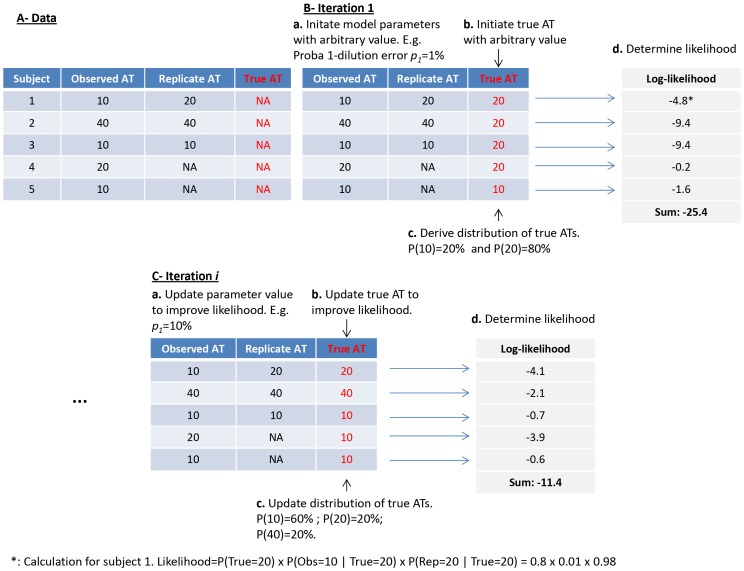
Less technical description of the statistical method. This figure illustrates the description of the method that is made in Box 1.

A less technical description of statistical methods is given for non-specialists in Box 1 and Figure 7.

**Box 1. Less-technical description of the statistical method**

In this box, we provide a less-technical description of the statistical method to give non-specialists an intuition of how it works. Readers should refer to the methods section for a technically rigorous description. From observed and replicate measurements of baseline and post epidemic ATs, our aim is to i) quantify measurement errors and ii) derive the *true* distribution of paired serology, that is, for example, to be able to estimate the *true* (*i.e.* after correction for measurement errors) proportion of subjects with ATs 10 at baseline and 40 post epidemic. For the sake of clarity, in this box, we restrict to the study of baseline ATs; but extending the approach to the joint analysis of baseline and post epidemic ATs is straightforward. We consider a toy dataset with 5 subjects with observed and replicate measurements for baseline ATs (Figure 7, panel A). Because of measurement errors, true baseline ATs are unknown (Figure 7, panel A). The statistical procedure is iterative. At iteration 1 (Figure 7, panel B), we start by initiating the model parameters and true ATs with arbitrary values (steps a and b). We can then derive the distribution of true ATs (step c) and calculate the probability (‘likelihood’) of the observed and replicate ATs given this initial set of parameters and characterisation of true ATs (step d). We are then running an iterative procedure called Markov chain Monte Carlo (MCMC) sampling. At each iteration (Figure 7, panel C) we are proposing new values for model parameters (step a) and for the true ATs of subjects (step b) in an attempt to improve the likelihood. After a certain number of iterations, parameters converge to the *posterior* distribution. This distribution gives likely values of parameters and also informs on uncertainty about those parameters. From the large sample of parameter values generated through 150,000 iterations of the MCMC procedure, we can calculate the posterior mean and 95% Credible Intervals (CI) of the parameters.

#### Notation

Antibody titers (AT) are discrete measurements that can take a finite number of values. In our dataset, they can take 9 values: *a_0_* = 10, *a_1_* = 20, *a_2_* = 40,…, *a_8_* = 2560, with the general form being *a_t_* = 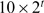 for *t* = 0,…,*K* (*K* = 8). For simplicity, in the rest of the paper, antibody titers are labelled by integer *t*. For example, AT level *t* = 0 corresponds to antibody titers *a_0_* = 10. 

We denote 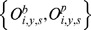 the “observed” AT levels measured at baseline (*b*) and post epidemic (*p*) in individual *i*, during season *y* ( = 2008, Spring 2009, Autumn 2009) and for subtype *s* ( =  H1N1, H3N2, B). In addition, for a subset of the blood samples, a replicate measurement of antibody titers was performed. We denote the replicate measurement for individual *i*, during season *y* and for subtype *s* (with *j = b* for baseline and *j = p* for post epidemic serology) by 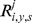 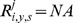 if no replicate measurement was performed.

Measurement errors mean that observed and replicate AT levels may be different from the true (but unobserved) AT levels that we denote by 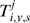.

#### Hierarchical structure of the statistical model

We build a 3-level Bayesian hierarchical model to characterize measurement errors together with the underlying true distribution of baseline and post-epidemic serology. The model is defined by the following equation:
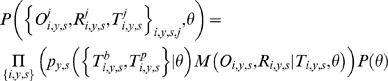
(1)where 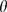 is the parameter vector of the model.

The first level 
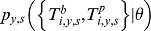
 of the model characterises the underlying true distribution of baseline and post-epidemic serology for each season and subtype. The second level 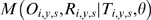
characterises measurement errors: given true AT levels 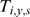, it gives the probability to measure 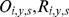 for the observed and replicate serology. The third level specifies our priors on model parameters. Each of those levels is described below, with more technical details given in the Supplementary Material.

#### Model for the underlying true serology

We consider the most general model for the joint distribution of true paired serology. For an individual *i*, during season *y* and for subtype *s*, each pair of serology measurements 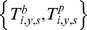
is drawn from a Multinomial distribution
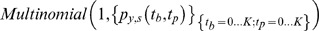
where 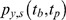
is the probability that 
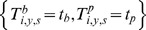
. We estimate these probabilities from the data.

#### Model for measurement errors

The quantity of antibodies in the blood of a subject can be thought of as a continuous variable. However, observations (*i.e.* AT titers) are discrete. We build a model of measurement errors that accounts for the continuous nature of the underlying biological variable. As mentioned earlier, AT measurements can take *K* values *T* = 0,…, *K*, corresponding to dilution levels of the HI assay. If the true (discrete) AT level is *T*, we assume that the continuous (unobserved) true quantity of antibodies in the blood, 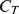, is uniformly distributed in the interval 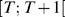. Conditional on the true quantity of antibodies 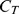, we introduce a function *f*(.) that indicates how far off from 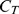 the observation can be: 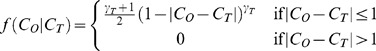
Conditional on true AT level *T* and on the titration not going wrong, the probability that the observed AT level is *O* is given by:
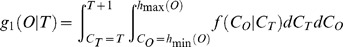
where 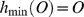 for O>0 and 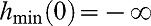; 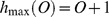 for O<K and 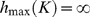 (NB: boundaries 0 and *K* are treated as special cases since data are truncated at those levels).

The probability of a 1-dilution (2-fold) error on one side (e.g. on the left) is 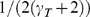.When the true AT level is not on the boundary 0 or *K*, the 2-sided probability of a 1-dilution error is 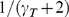.

The joint probability for the pair 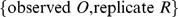 is:
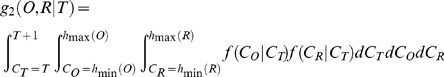
We also assume that there is a probability *ε* that the titration goes wrong and the resulting titre measurement is an integer uniformly drawn from 0 to *K*. Conditional on true AT levels *T*, the probability distribution for *O* is therefore:
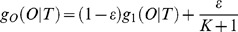
and the joint probability for the pair 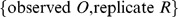 is:
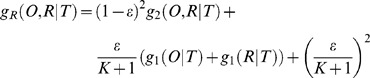



#### Prior model

For each season *y* and subtype *s*, we assume that the set of probabilities 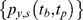 characterizing true paired serology has a Dirichlet prior distribution 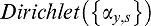, where hyperparameter 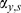 has a uniform hyperprior distribution on [0, 1000] (see Supplementary Material). The Dirichlet distribution is the conjugate prior of the multinomial distribution. Other parameters of the model have uniform priors.

#### Data augmentation and inference

True AT levels 
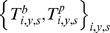
 are considered as augmented data and a Markov chain Monte Carlo (MCMC) sampling algorithm is used to explore the joint distribution of augmented data and parameters [26]. At each iteration of the MCMC, the following updates, which are detailed in the Supplementary Material, are implemented:

Update 1: For each subject *i*, season *y*, subtype *s*, independence sampler for true AT levels 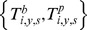;
Update 2: For each season *y* and subtype *s*, Gibbs sampler for the probability distribution of paired serology 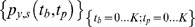;
Update 3: For each season *y* and subtype *s*, Metropolis-Hastings update of hyperparameter 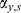;
Update 4: Metropolis-Hastings update of parameters characterizing measurement errors.

Information on measurement errors is contained in the data from the subset of individuals for whom a replicate measurement was performed. If update 4 (on measurement error parameters) was run on the full likelihood, the inference would suffer a “feedback” problem, with estimates of measurement errors being potentially largely driven by the larger (yet poorly informative) subset of individuals for whom no replicate measurements are available. We therefore use a standard strategy to circumvent this problem that consists in only using the contribution of individuals with replicate measurements in update 4 (see for example, function “cut” in WinBugs) [27]–[29]. Technical details are given in the Supplementary Material.

#### Selection of subjects for whom replicate measurements were performed

The subjects for whom replicate measurements were performed were not selected at random (Table S1). For example, those that had low antibody titers at baseline and post epidemic were never selected. To correct for this selection bias we model the selection process and make estimation of parameters characterizing measurement errors conditional on those individuals being selected. Technical details are given in the Supplementary Material.

#### Simulation study

In order to assess the performance of the method to quantify measurement errors and reconstruct the true distribution of paired serology, a simulation study is implemented. Eighty datasets with a structure similar to ours (i.e. same number of subtype/season, same number of observed paired serology per subtype/season) are simulated from the posterior mean of the parameters and the distribution of the true paired serology. The selection of subjects for whom replicate measurements are performed is simulated as in our model. We then applied our statistical model to each of the simulated datasets and assessed the bias on parameters quantifying measurement errors and on the true distribution of paired serology.

## Ethics statement

The research was approved by the institutional review board of the National Institute of Hygiene and Epidemiology, Vietnam; the Oxford Tropical Research Ethics Committee, University of Oxford, UK; and the Ethics Committee of the London School of Hygiene and Tropical Medicine, UK. All participants provided written informed consent.

## Supporting Information

Figure S1 **Fit of the model where measurement errors are independent of true antibody titers to data on replicate measurements.** Observed (red point) and expected (mean: blue point/95% CI: blue bar) number of pairs {observed AT level, replicate AT level}. Pairs are sorted by panel according to the number of dilution difference between the observed and the replicate measurement.
(EPS)

Figure S2 **Adequacy of model where measurement errors are independent of true antibody titers to the data.** Distribution of “observed” paired serology as predicted by the model (color bars) and as observed in the data (black point) as a function of season (2008, Spring 2009, Autumn 2009) and subtype (H1N1, H3N2 and B). In each panel, individuals are sorted by baseline AT levels on the y-axis. For a given baseline, the grey bar indicates the expected proportion of individuals with post AT level equal to baseline AT level; the yellow bar indicates the proportion with a 2 fold rise (2f.r.); the red bar indicates the proportion with a 4 fold rise or more (4f.r.+); the green bar indicates the proportion with a decay. The black thin lines give the 95% CI. The legend gives the mean [95% CI]. **A**: H1N1, 2008. **B**: H3N2, 2008. **C**: B, 2008. **D**: H1N1, Spring 2009. **E**: H3N2, Spring 2009. **F**: B, Spring 2009. **G**: H1N1pdm09, Autumn 2009.
(EPS)

Figure S3 **Testing the absence of cross-reactivity between subtypes.** For each year and each subtype, individuals were partitioned between those with no increase in titers (coded 0), those with a 1-dilution increase (coded 1) and those with a 2 dilution or more increase (coded 2). The population was then partitioned in 27 groups according to outcome for triplet H1N1-H3N2-B. For example triplet 1-0-0 consists of individuals with a 1-dilution increase for H1N1 but no increase for H3N2 and B; 1-2-0 are individuals with a 1-dilution increase for H1N1, 2-dilution increase for H3 but no increase for B etc. Red points show the mean posterior distribution for triplet H1N1-H3N2-B, corrected for measurement errors. The boxplots in the figure show the distribution that would be obtained if there was no cross-reactivity between subtypes.
(EPS)

Figure S4 **Age-specific distribution of paired serology, corrected for measurement errors as a function of season (2008, Spring 2009, Autumn 2009) and subtype (H1N1, H3N2 and B) (in Autumn 2009, subtyping was only conducted for H1N1pdm09).** In each panel, individuals are sorted by baseline AT levels on the y-axis. For a given baseline, the grey bar indicates the expected proportion of individuals with post AT level equal to baseline AT level; the yellow bar indicates the proportion with a 2 fold rise (2f.r.); the red bar indicates the proportion with a 4 fold rise or more (4f.r.+); the green bar indicates the proportion with a decay. The black thin lines give the 95% CI. The legend gives the mean [95% CI]. **A**: H1, 2008, <18 y.o. **B**: H3, 2008, <18 y.o. **C**: B, Spring 2009, <18 y.o. **D**: H1, 2008, <18 y.o. **E**: H3, Spring 2009, <18 y.o. **F**: B, Spring 2009, <18 y.o. **G**: H1pdm, Autumn 2009, <18 y.o. **H**: H1, 2008, 18–48 y.o. **I**: H3, 2008, 18–48 y.o. **J**: B, Spring 2009, 18–48 y.o. **K**: H1, 2008, 18–48 y.o. **L**: H3, Spring 2009, 18–48 y.o. **M**: B, Spring 2009, 18–48 y.o. **N**: H1pdm, Autumn 2009, 18–48 y.o. **O**: H1, 2008, >48 y.o. **P**: H3, 2008, >48 y.o. **Q**: B, Spring 2009, >48 y.o. **R**: H1, 2008, >48 y.o. **S**: H3, Spring 2009, >48 y.o. **T**: B, Spring 2009, >48 y.o. **U**: H1pdm, Autumn 2009, >48 y.o.
(EPS)

Figure S5 **Model adequacy to age-specific data.** Distribution of “observed” paired serology as predicted by the model (color bars) and as observed in the data (black point) as a function of season (2008, Spring 2009, Autumn 2009), subtype (H1N1, H3N2 and B) and age group (<18 y.o., 18–48 y.o., >48 y.o.). In each panel, individuals are sorted by baseline AT levels on the y-axis. For a given baseline, the grey bar indicates the expected proportion of individuals with post AT level equal to baseline AT level; the yellow bar indicates the proportion with a 2 fold rise (2f.r.); the red bar indicates the proportion with a 4 fold rise or more (4f.r.+); the green bar indicates the proportion with a decay. The black thin lines give the 95% CI. The legend gives the mean [95% CI]. **A**: H1, 2008, <18 y.o. **B**: H3, 2008, <18 y.o. **C**: B, Spring 2009, <18 y.o. **D**: H1, 2008, <18 y.o. **E**: H3, Spring 2009, <18 y.o. **F**: B, Spring 2009, <18 y.o. **G**: H1pdm, Autumn 2009, <18 y.o. **H**: H1, 2008, 18–48 y.o. **I**: H3, 2008, 18–48 y.o. **J**: B, Spring 2009, 18–48 y.o. **K**: H1, 2008, 18–48 y.o. **L**: H3, Spring 2009, 18–48 y.o. **M**: B, Spring 2009, 18–48 y.o. **N**: H1pdm, Autumn 2009, 18–48 y.o. **O**: H1, 2008, >48 y.o. **P**: H3, 2008, >48 y.o. **Q**: B, Spring 2009, >48 y.o. **R**: H1, 2008, >48 y.o. **S**: H3, Spring 2009, >48 y.o. **T**: B, Spring 2009, >48 y.o. **U**: H1pdm, Autumn 2009, >48 y.o.
(EPS)

Figure S6 **Distribution of observed paired serology in** [**23**] **.** **A**: HI assay for seasonal H1N1 influenza (2009). **B**: Micro-neutralization assay for pandemic H1N1 influenza (2009). **C**: HI assay for pandemic A(H1N1)pdm09 influenza (2009).
(EPS)

Table S1
**Probability (numerator/denominator) that replicate measurements are performed during 2008 and Spring 2009 seasons, for subtype H1N1, as a function of observed serology at baseline and post epidemic.** The colors indicate how we model the probability of selection. Yellow cells correspond to cells for which we assume that the probability of selection is null. The probabilities associated to the 4 other colors are estimated from the data (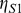: orange ; 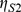: red; 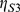: light green; 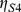: green). See Supplementary Material for details.(DOCX)

Table S2 **Performance of the method to estimate parameters characterizing how subjects with duplicate measurements were selected.** Those parameters are defined in Table S1 (see also section 1 of Supplementary Material). Eighty datasets are simulated with known parameters (see Methods). The table gives the simulation value of parameters and the mean (standard deviation) of estimates.
(DOCX)

Text S1 **Technical details on the model, the estimation procedure, sensitivity analyses, and the test for the hypothesis of cross-reactivity between subtypes.**
(DOCX)
