# Supplementary material for: Influenza Infection Rates, Measurement Errors and the Interpretation of Paired Serology
Source: PLoS Pathog. 2012 Dec 13;8(12):e1003061. doi: 10.1371/journal.ppat.1003061 (PMC3521724; doi:10.1371/journal.ppat.1003061)
Supplement: Table S1 — Probability (numerator/denominator) that replicate measurements are performed during 2008 and Spring 2009 seasons, for subtype H1N1, as a function of observed serology at baseline and post epidemic. The colors indicate how we model the probability of selection. Yellow cells correspond to cells for which we assume that the probability of selection is null. The probabilities associated to the 4 other colors are estimated from the data (: orange ; : red; : light green; : green). See Supplementary Material for details. (DOCX) [file ppat.1003061.s007.docx]

**Table S1**

|  |  | **Antibody titers levels post-epidemic** | | | | | | | | |
| --- | --- | --- | --- | --- | --- | --- | --- | --- | --- | --- |
| **2008** | | 0 | 1 | 2 | 3 | 4 | 5 | 6 | 7 | 8 |
| **Antibody titers levels at baseline** | 0 | 0% (0/214) | 0% (0/117) | 0% (0/22) | 100% (12/12) | 100% (10/10) | 100% (3/3) | 100% (1/1) | NA (0/0) | NA (0/0) |
|  | 1 | 0% (0/32) | 0% (0/48) | 0% (0/22) | 75% (3/4) | 100% (1/1) | 100% (1/1) | NA (0/0) | NA (0/0) | NA (0/0) |
|  | 2 | 0% (0/2) | 0% (0/10) | 0% (0/13) | 63% (5/8) | 100% (2/2) | 100% (1/1) | NA (0/0) | NA (0/0) | NA (0/0) |
|  | 3 | 100% (2/2) | 100% (1/1) | 0% (0/9) | 0% (0/6) | 100% (3/3) | 100% (1/1) | NA (0/0) | NA (0/0) | NA (0/0) |
|  | 4 | 100% (1/1) | NA (0/0) | 100% (1/1) | 100% (2/2) | 100% (1/1) | NA (0/0) | NA (0/0) | NA (0/0) | NA (0/0) |
|  | 5 | NA (0/0) | NA (0/0) | NA (0/0) | NA (0/0) | NA (0/0) | NA (0/0) | NA (0/0) | NA (0/0) | NA (0/0) |
|  | 6 | NA (0/0) | NA (0/0) | NA (0/0) | NA (0/0) | NA (0/0) | NA (0/0) | NA (0/0) | NA (0/0) | NA (0/0) |
|  | 7 | NA (0/0) | NA (0/0) | NA (0/0) | NA (0/0) | NA (0/0) | NA (0/0) | NA (0/0) | NA (0/0) | NA (0/0) |
|  | 8 | NA (0/0) | NA (0/0) | NA (0/0) | NA (0/0) | NA (0/0) | NA (0/0) | NA (0/0) | NA (0/0) | NA (0/0) |
|  |  |  |  |  |  |  |  |  |  |  |
| **Spring 2009** | | 0 | 1 | 2 | 3 | 4 | 5 | 6 | 7 | 8 |
| **Antibody titers levels at baseline** | 0 | 0% (0/247) | 0% (0/63) | 0% (0/23) | 50% (5/10) | 86% (6/7) | 100% (7/7) | 100% (2/2) | NA (0/0) | NA (0/0) |
|  | 1 | 0% (0/11) | 0% (0/23) | 0% (0/13) | 75% (3/4) | NA (0/0) | 100% (1/1) | NA (0/0) | 100% (6/6) | NA (0/0) |
|  | 2 | 0% (0/3) | 0% (0/11) | 0% (0/18) | 0% (0/12) | 80% (4/5) | 100% (3/3) | NA (0/0) | NA (0/0) | NA (0/0) |
|  | 3 | 0% (0/1) | NA (0/) | 0% (0/6) | 0% (0/11) | 0% (0/1) | 100% (1/1) | NA (0/0) | 100% (1/1) | NA (0/0) |
|  | 4 | NA (0/0) | NA (0/0) | NA (0/0) | 0% (0/5) | 0% (0/6) | NA (0/0) | NA (0/0) | NA (0/0) | NA (0/0) |
|  | 5 | NA (0/0) | NA (0/0) | NA (0/0) | NA (0/0) | 0% (0/2) | 0% (0/1) | NA (0/0) | NA (0/0) | NA (0/0) |
|  | 6 | NA (0/0) | NA (0/0) | NA (0/0) | NA (0/0) | NA (0/0) | 0% (0/1) | NA (0/0) | NA (0/0) | NA (0/0) |
|  | 7 | NA (0/0) | NA (0/0) | NA (0/0) | NA (0/0) | NA (0/0) | NA (0/0) | NA (0/0) | NA (0/0) | NA (0/0) |
|  | 8 | NA (0/0) | NA (0/0) | NA (0/0) | NA (0/0) | NA (0/0) | NA (0/0) | NA (0/0) | NA (0/0) | NA (0/0) |
