# Supplementary material for: Influenza Infection Rates, Measurement Errors and the Interpretation of Paired Serology
Source: PLoS Pathog. 2012 Dec 13;8(12):e1003061. doi: 10.1371/journal.ppat.1003061 (PMC3521724; doi:10.1371/journal.ppat.1003061)
Supplement: Table S2 — Performance of the method to estimate parameters characterizing how subjects with duplicate measurements were selected. Those parameters are defined in Table S1 (see also section 1 of Supplementary Material). Eighty datasets are simulated with known parameters (see Methods). The table gives the simulation value of parameters and the mean (standard deviation) of estimates. (DOCX) [file ppat.1003061.s008.docx]

**Table S2**

|  | ***$\eta_{S1}$*** | ***$\eta_{S2}$*** | ***$\eta_{S3}$*** | ***$\eta_{S4}$*** |
| --- | --- | --- | --- | --- |
| **Simulation value** | 82.0% | 98.0% | 56.0 | 95.0% |
| **Mean estimate (SD)** | 81.5% (8.6%) | 96.2% (2.5%) | 55.9% (16.1%) | 92.1% (4.3%) |
