# Supplementary material for: Influenza Infection Rates, Measurement Errors and the Interpretation of Paired Serology
Source: PLoS Pathog. 2012 Dec 13;8(12):e1003061. doi: 10.1371/journal.ppat.1003061 (PMC3521724; doi:10.1371/journal.ppat.1003061)
Supplement: Text S1 — Technical details on the model, the estimation procedure, sensitivity analyses, and the test for the hypothesis of cross-reactivity between subtypes. (DOCX) [file ppat.1003061.s009.docx]

**Supplementary Information**

**Influenza infection rates, measurement errors and the interpretation of paired serology**

Simon Cauchemez^1^, Peter Horby^2^, Annette Fox^2^, Le Quynh Mai^3^, Le Thi Thanh^3^, Pham Quang Thai^3^, Le Nguyen Minh Hoa^2^, Nguyen Tran Hien^3^, Neil M. Ferguson^1^

1 MRC Centre for Outbreak Analysis and Modelling, Department of Infectious Disease Epidemiology, Imperial College London, London, United Kingdom,

2 Oxford University Clinical Research Unit - Wellcome Trust Major Overseas Programme, Hanoi, Vietnam,

3 National Institute of Hygiene and Epidemiology, Hanoi, Vietnam

**Contents**

[1 Selection of subjects from whom replicate measurements were performed and model for measurement errors 2](#_Toc337467184)

[1.1 Selection in the study 2](#_Toc337467185)

[1.2 Model 2](#_Toc337467186)

[2 Joint distribution of parameters and augmented data 3](#_Toc337467187)

[3 MCMC 3](#_Toc337467188)

[3.1 MCMC Updates 3](#_Toc337467189)

[3.1.1 Update 1: individual true AT levels 3](#_Toc337467190)

[3.1.2 Update 2: updating the distribution of “true” paired serology 4](#_Toc337467191)

[3.1.3 Update 3: Hyperparameters$\left\{\alpha_{y,s}\right\}$ 4](#_Toc337467192)

[3.1.4 Update 4: Parameters characterizing measurement errors 4](#_Toc337467193)

[3.2 Implementation 4](#_Toc337467194)

# Selection of subjects from whom replicate measurements were performed and model for measurement errors

## Selection in the study

Replicate measurements were performed for a subset of individuals during 2008 and Spring 2009 seasons, for subtype H1N1. Table S1 shows how the probability to have replicate measurements being performed depended on observed serology at baseline and post epidemic. Looking at those tables, it is clear that the subjects for whom replicate measurements were performed were not selected at random. For example, those that had low antibody titers at baseline and post epidemic were never selected.

## Model

To avoid a potential selection bias, we account for this selection process in our inferential framework. For season *y*=2008 or Spring 2009 and subtype *s*=H1N1, the contribution of individual *i* to the measurement error model is:

- If replicate measurements were performed for the individual:

$$M\left(O_{i,y,s},R_{i,y,s}|T_{i,y,s},\theta\right)=P_{S,y}\left(O_{i,y,s}^{b},O_{i,y,s}^{p}|\theta\right)\prod_{j=\left\{b,p\right\}} g_{R}\left(O_{i,y,s}^{j},R_{i,y,s}^{j}|T_{i,y,s}^{j},\theta\right)$$

where$P_{S,y}\left(O_{i,y,s}^{b},O_{i,y,s}^{p}|\theta\right)$ is the probability that replicate measurements are performed as a function of observed AT levels. This probability can take 5 different values (0, and 4 values that are estimated from the data) (see Table S1).

- If no replicate measurement was performed for individual *i*:

$$M\left(O_{i,y,s},R_{i,y,s}|T_{i,y,s},\theta\right)=\left(1-P_{S,y}\left(O_{i,y,s}^{b},O_{i,y,s}^{p}|\theta\right)\right)\prod_{j=\left\{b,p\right\}} g_{O}\left(O_{i,y,s}^{j}|T_{i,y,s}^{j},\theta\right)$$

For other seasons/subtypes, the measurement error model is simply:

$$M\left(O_{i,y,s},R_{i,y,s}|T_{i,y,s},\theta\right)=\prod_{j=\left\{b,p\right\}} g_{O}\left(O_{i,y,s}^{j}|T_{i,y,s}^{j},\theta\right)$$

# Joint distribution of parameters and augmented data

We specify a Dirichlet prior distribution$Dirichlet\left(\left\{\alpha_{y,s}\right\}\right)$ for distribution$\left\{p_{y,s}\left(t_{b},t_{p}\right)\right\}$:

$$P\left(\left\{p_{y,s}\left(k_{b},k_{p}\right)\right\}|\alpha_{y,s}\right)=\frac{\Gamma\left(K^{2}\alpha_{y,s}\right)}{\Gamma\left(\alpha_{y,s}\right)^{K^{2}}}\prod_{t_{b},t_{p}} p_{y,s}\left(t_{b},t_{p}\right)^{\alpha_{y,s}-1}$$

where hyperparameter$\alpha_{y,s}$has a Uniform([0,1000]) hyperprior distribution.

Denote$n_{y,s}\left(t_{b},t_{p}\right)$the number of subjects with true paired serology $\left\{t_{b},t_{p}\right\}$during season *y* and for subtype *s*

$$n_{y,s}\left(t_{b},t_{p}\right)=\sum_{i} \mathbf{1}\left(T_{i,y,s}^{b}=t_{b},T_{i,y,s}^{p}=t_{p}\right)$$

The joint distribution of parameters and augmented data can be re-written:

(S1)

# MCMC

## MCMC Updates

### Update 1: individual true AT levels

At each iteration of the MCMC, for each season *y* and subtype *s*, we update true AT levels at baseline and post epidemic, for $2M_{y,s}$randomly selected individuals, where $M_{y,s}$is the number of individuals with serology available for season *y* and subtype *s*. Consider such an individual i, with observed and replicate measurements $\left\{o_{b},o_{p},r_{b},r_{p}\right\}$and with true AT levels $\left\{t_{b},t_{p}\right\}$at iteration *n*. At iteration *n+1*, we use an independence sampler to simultaneously propose new candidate values $\left\{t_{b}^{*},t_{p}^{*}\right\}$for true AT levels.

For *j=b,p*, the new candidate $t_{j}^{*}$is drawn from a multinomial distribution $Multinomial\left(1,Q_{o_{j}}\right)$, where $Q_{o_{j}}$corresponds to column *o_j_* of matrix *Q*:

$$Q=\left[\begin{matrix} 66\% & 25\% & 6\% & 0.5\% & 0.5\% & 0.5\% & 0.5\% & 0.5\% & 0.5\% \\ 25\% & 36\% & 25\% & 6\% & 0.5\% & 0.5\% & 0.5\% & 0.5\% & 0.5\% \\ 6\% & 25\% & 36\% & 25\% & 6\% & 0.5\% & 0.5\% & 0.5\% & 0.5\% \\ 0.5\% & 6\% & 25\% & 36\% & 25\% & 6\% & 0.5\% & 0.5\% & 0.5\% \\ 0.5\% & 0.5\% & 6\% & 25\% & 36\% & 25\% & 6\% & 0.5\% & 0.5\% \\ 0.5\% & 0.5\% & 0.5\% & 6\% & 25\% & 36\% & 25\% & 6\% & 0.5\% \\ 0.5\% & 0.5\% & 0.5\% & 0.5\% & 6\% & 25\% & 36\% & 25\% & 6\% \\ 0.5\% & 0.5\% & 0.5\% & 0.5\% & 0.5\% & 6\% & 25\% & 36\% & 25\% \\ 0.5\% & 0.5\% & 0.5\% & 0.5\% & 0.5\% & 0.5\% & 6\% & 25\% & 66\% \end{matrix}\right]$$

### Update 2: updating the distribution of “true” paired serology

A Gibbs sampler [[1](#_ENREF_1)] is developed to update$\left\{p_{y,s}\left(t_{b},t_{p}\right)\right\}$. The full conditional distribution is simply:

$$Dirichlet\left(\left\{n_{y,s}\left(t_{b},t_{p}\right)+\alpha_{y,s}\right\}_{t_{b},t_{p}}\right)$$

### Update 3: Hyperparameters$\left\{\alpha_{y,s}\right\}$

This is performed with a standard Metropolis-Hastings algorithm based on equation (S1) [[1](#_ENREF_1)].

### Update 4: Parameters characterizing measurement errors

As explained in the main text, in order to avoid a “feedback” problem (whereby measurement errors estimates are driven by the larger _yet poorly informative_ subset of individuals for whom no replicate measurement was performed), only individuals with replicate measurements contribute to the update of parameters with measurement errors in the MCMC [[2-4](#_ENREF_2)]. To correct for the selection bias described in section 1 of the Supplementary Material, the contribution of such individual *i*, for season *y* (= 2008, or Spring 2009) and subtype *s* (=H1N1) is conditional on the fact that the individual was selected (for a replicate measurement to be performed):

$$\frac{M\left(O_{i,y,s},R_{i,y,s}|T_{i,y,s},\theta\right)}{P_{\text{Select},y}\left(\theta\right)}$$

where the probability of being selected is the sum

$$P_{\text{Select},y}\left(\theta\right)=\sum_{k_{b},k_{p},o_{b},o_{p}} p_{y,s}\left(k_{b},k_{p}\right)g_{O}\left(o_{b}|k_{b},\theta\right)g_{O}\left(o_{p}|k_{p},\theta\right)P_{S,y}\left(o_{b},o_{p}|\theta\right)$$

(the first term corresponds to the probability associated with the true distribution; the second and third terms correspond to measurement errors; the last term corresponds to the probability of selection given observed serology).

## Implementation

The chain is run for 150,000 iterations with a burn in of 5,000 and a thinning of 10.

Model where measurement errors are independent of true AT level

We also considered the model where the probability of measurement error was the same for AT level =0 and for AT levels >0. Under this assumption, the 1-sided probability of a 1-dilution error was 17.9 (95% CI: 13.7%, 21.8%). The fit of this model to data on duplicates remains good (Figure S1). However, the fit to the observed distribution of paired serology is bad (Figure S2):

- The model underestimates the proportion of individuals with observed paired serology {baseline AT=0, post AT=0} for H3N2 in 2008, H1N1 in Spring 2009, H1N1pdm09 in Autumn 2009.
- The model overestimates the proportion of individuals with observed paired serology {baseline AT=1; post AT=0} for H3N2 in 2008, H1N1 and H3N2 in Spring 2009, H1N1pdm09 in Autumn 2009.
- The model underestimates the proportion of individuals with observed paired serology {baseline AT=0, post AT=1} for H1N1pdm09 in Autmn 2009.

Testing the hypothesis of cross-reactivity between subtypes

We explored whether there was significant cross-reactivity between seasonal subtypes H1N1, H3N2 and B in 2008 and Spring 2009. First, for each season and each subtype, individuals were partitioned between those with no increase in titers (coded 0), those with a 1-dilution increase (coded 1) and those with a 2 dilution or more increase (coded 2). The population was then partitioned in 27 groups according to outcome for triplet H1N1-H3N2-B. For example triplet 1-0-0 consists of individuals with a 1-dilution increase for H1N1 but no increase for H3N2 and B; 1-2-0 are individuals with a 1-dilution increase for H1N1, 2-dilution increase for H3 but no increase for B etc.

Red points in Figure S3 show the mean posterior distribution for triplet H1N1-H3N2-B, corrected for measurement errors. The boxplots in the figure show the distribution that would be obtained if there was no cross-reactivity between subtypes. This is obtained by permutating subtype-specific status among subjects, independently for each subtype. Three thousand datasets were generated that way. There is a good adequacy between the observed distribution and the “no cross-reactivity” distribution (observed frequencies are all within the 95% CI of the “no cross-reactivity” distribution). We cannot reject the assumption of an absence of cross-reactivity between subtypes.

Bibliography

1. Gilks WR, Richardson S, Spiegelhalter DJ (1996) Markov Chain Monte Carlo in Practice. London: Chapman and Hall.

2. Best N, Spiegelhalter DJ, Lunn D, Graham G, Neuenschwander B (2006) Cutting "feedback" in Bayesian full probability models. A workshop about the development and use of the BUGS programme. Hanko, Finland. Available at <http://mathstat.helsinki.fi/openbugs/IceBUGS/Presentations/BestIceBUGS.pdf>. Accessed on 22 June 2012.

3. Erasto P, Hoti F, Auranen K (2012) Modeling transmission of multitype infectious agents: application to carriage of Streptococcus pneumoniae. Statistics in Medicine 31: 1450-1463.

4. Spiegelhalter DJ, Thomas A, Best N, Lunn D (2003) WinBUGS User Manual Version 1.4, 2003. Available at <http://www.mrc-bsu.cam.ac.uk/bugs/winbugs/manual14.pdf>. Accessed on 22 June 2012.

5. Cowling BJ, Chan KH, Fang VJ, Lau LL, So HC, et al. (2010) Comparative epidemiology of pandemic and seasonal influenza A in households. New England Journal of Medicine 362: 2175-2184.
